# (1S*,3R*,5S*,7S*)-4,4,8,8-Tetra­chloro-1-isopropyl-5-methyl­tri­cyclo­[5.1.0.0^3,5^]octa­ne

**DOI:** 10.1107/S1600536814004826

**Published:** 2014-03-12

**Authors:** Koblandy M. Turdybekov, Oleg G. Ryazantsev, Gayane A. Atazhanova, Sergazy M. Adekenov

**Affiliations:** a International Reseach and Production Holding "Phytochemistry", Gazaliev St 4, 100009 Karaganda, Kazakhstan

## Abstract

The title compound, C_12_H_16_Cl_4_, is a derivative of the natural product 1-isopropyl-4-methyl­cyclo­hexa-1,4-diene, and represents a diastereomer with two *trans*-fused cyclo­propane rings. Both enanti­omers are present in the non-centrosymmetric polar space group *Pna*2_1_. The central cyclo­hexane ring is planar within 0.02 (1) Å. The C atoms of di­chloro­methyl­ene groups deviate from this plane by 1.19 (1) and −1.26 (1) Å, whereas the isopropyl and methyl groups are oriented more equatorially, deviating by 0.71 (1) and −0.87 (1) Å, respectively.

## Related literature   

For the isolation of 1-isopropyl-4-methyl­cyclo­hexa-1,4-diene, see: Jamali *et al.* (2013 ▶). For the crystal structure of a related compound, see: Lynch *et al.* (1994 ▶).
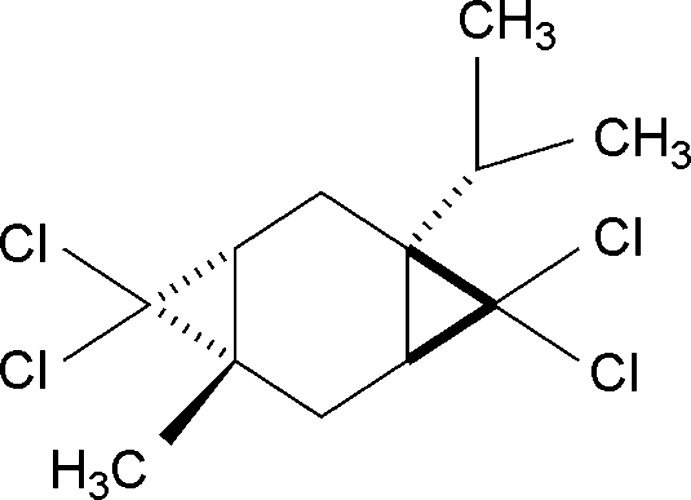


## Experimental   

### 

#### Crystal data  

C_12_H_16_Cl_4_
*M* *_r_* = 302.05
Orthorhombic, 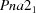

*a* = 10.9480 (3) Å
*b* = 11.8207 (3) Å
*c* = 10.5027 (4) Å
*V* = 1359.19 (7) Å^3^
*Z* = 4
Mo *K*α radiation
μ = 0.84 mm^−1^
*T* = 150 K
0.30 × 0.26 × 0.02 mm

#### Data collection  

Bruker APEXII CCD diffractometer
Absorption correction: multi-scan (*SADABS*; Bruker, 2008 ▶) *T*_min_ = 0.786, *T*_max_ = 0.988
9762 measured reflections
3130 independent reflections
2973 reflections with *I* > 2σ(*I*)
*R*_int_ = 0.021

#### Refinement  

*R*[*F* ^2^ > 2σ(*F* ^2^)] = 0.023
*wR*(*F* ^2^) = 0.054
*S* = 1.05
3130 reflections
148 parameters
1 restraint
H-atom parameters constrained
Δρ_max_ = 0.23 e Å^−3^
Δρ_min_ = −0.16 e Å^−3^
Absolute structure: Flack (1983 ▶), 1313 Friedel pairs
Absolute structure parameter: 0.09 (4)

### 

Data collection: *APEX2* (Bruker, 2005 ▶); cell refinement: *SAINT* (Bruker, 2005 ▶); data reduction: *SAINT* (Bruker, 2005 ▶); program(s) used to solve structure: *SHELXS97* (Sheldrick, 2008 ▶); program(s) used to refine structure: *SHELXL97* (Sheldrick, 2008 ▶); molecular graphics: *SHELXTL* (Sheldrick, 2008 ▶); software used to prepare material for publication: *SHELXTL* (Sheldrick, 2008 ▶).

## Supplementary Material

Crystal structure: contains datablock(s) global, I. DOI: 10.1107/S1600536814004826/ld2121sup1.cif

Structure factors: contains datablock(s) I. DOI: 10.1107/S1600536814004826/ld2121Isup2.hkl

Supporting information file. DOI: 10.1107/S1600536814004826/ld2121Isup3.cdx

Supporting information file. DOI: 10.1107/S1600536814004826/ld2121Isup4.cml

CCDC reference: 989552

Additional supporting information:  crystallographic information; 3D view; checkCIF report

## Figures and Tables

**Fig. 1.**
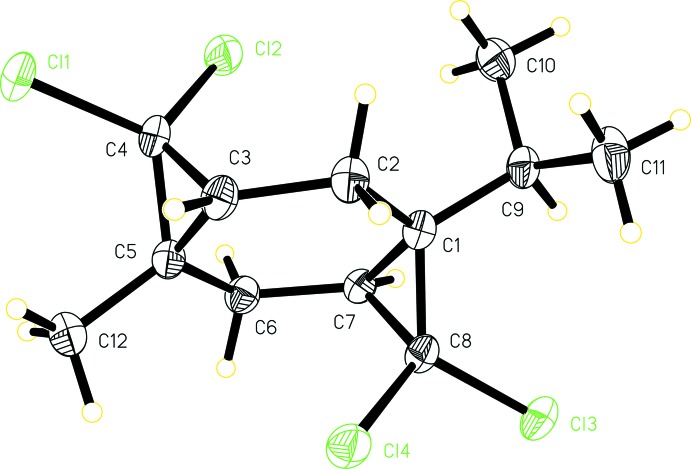
The molecular structure of (I), with atom labels and 50% probability displacement ellipsoids for non-H atoms.

## supplementary crystallographic information

### 1. Comment

The molecule of the title compound, (I) (Fig.1) is an enantiomeric pair of diastereomers. The relative configuration at positions 1, 3, 5 and 7 was established as S, R, S, and S, respectively. The cyclohexane ring is approximately planar, the maximum deviation from the mean plane being 0.02 (1) Å. The atoms C4 and C8 of cyclopentane rings deviate from this plane on 1.19 (1) and -1.26 (1) Å, atom C9 of isopropyl and atom C12 of methyl groups deviate on 0.71 (1) and -0.87 (1) Å, respectively.

### 2. Experimental

The title compound was synthesized by interaction of /g-terpinene 1-isopropyl-4-methylcyclohexa-1,4-diene, which was isolated from the essential oil of above aerial part of *Juniperus sabina L*., with NaOH in CHCl_3_ in presence of triethylbenzylammonium chloride with yield 72% and with melting point 80–84°C.

### 3. Refinement

H atoms were positioned geometrically and refined using a riding and rotating model, with C—H = 0.98–1.0 Å and with *U*_iso_(H) = 1.2 (1.5 for methyl groups) times the *U*_eq_(C). The absolute configurations of the crystal was established by refinement of the Flack parameter.

### Crystal data

**Table:**

C_12_H_16_Cl_4_	*D*_x_ = 1.476 Mg m^−^^3^
*M**_r_* = 302.05	Melting point = 357–353 K
Orthorhombic, *P**n**a*2_1_	Mo *K*α radiation, λ = 0.71073 Å
Hall symbol: P 2c -2n	Cell parameters from 5371 reflections
*a* = 10.9480 (3) Å	θ = 2.5–29.2°
*b* = 11.8207 (3) Å	µ = 0.84 mm^−^^1^
*c* = 10.5027 (4) Å	*T* = 150 K
*V* = 1359.19 (7) Å^3^	Irregular, colourless
*Z* = 4	0.30 × 0.26 × 0.02 mm
*F*(000) = 624	

### Data collection

**Table:**

Bruker APEXII CCD diffractometer	3130 independent reflections
Radiation source: fine-focus sealed tube	2973 reflections with *I* > 2σ(*I*)
Graphite monochromator	*R*_int_ = 0.021
Detector resolution: 7.11 pixels mm^-1^	θ_max_ = 29.7°, θ_min_ = 2.6°
φ and ω scans	*h* = −13→13
Absorption correction: multi-scan (*SADABS*; Bruker, 2008)	*k* = −13→15
*T*_min_ = 0.786, *T*_max_ = 0.988	*l* = −14→13
9762 measured reflections	

### Refinement

**Table:**

Refinement on *F*^2^	Secondary atom site location: difference Fourier map
Least-squares matrix: full	Hydrogen site location: inferred from neighbouring sites
*R*[*F*^2^ > 2σ(*F*^2^)] = 0.023	H-atom parameters constrained
*wR*(*F*^2^) = 0.054	*w* = 1/[σ^2^(*F*_o_^2^) + (0.0259*P*)^2^ + 0.1704*P*] where *P* = (*F*_o_^2^ + 2*F*_c_^2^)/3
*S* = 1.05	(Δ/σ)_max_ = 0.001
3130 reflections	Δρ_max_ = 0.23 e Å^−^^3^
148 parameters	Δρ_min_ = −0.16 e Å^−^^3^
1 restraint	Absolute structure: Flack (1983), 1313 Friedel pairs
Primary atom site location: structure-invariant direct methods	Absolute structure parameter: 0.09 (4)

### Fractional atomic coordinates and isotropic or equivalent isotropic displacement parameters (Å^2^)

**Table:**

	*x*	*y*	*z*	*U*_iso_*/*U*_eq_	
Cl1	0.57150 (4)	−0.08034 (4)	1.00600 (5)	0.03549 (12)	
Cl2	0.38725 (4)	0.05915 (4)	1.12573 (5)	0.03067 (11)	
Cl3	−0.09617 (3)	0.02606 (4)	0.84836 (4)	0.02866 (11)	
Cl4	0.10624 (4)	−0.10256 (4)	0.74584 (4)	0.02984 (11)	
C1	0.15053 (13)	0.08350 (13)	0.90807 (15)	0.0188 (3)	
C2	0.27707 (13)	0.08167 (13)	0.84709 (17)	0.0232 (3)	
H2A	0.2665	0.0869	0.7537	0.028*	
H2B	0.3213	0.1504	0.8748	0.028*	
C3	0.35723 (14)	−0.01975 (14)	0.87507 (16)	0.0228 (3)	
H3	0.4093	−0.0434	0.8015	0.027*	
C4	0.41698 (13)	−0.03625 (14)	1.00155 (18)	0.0240 (3)	
C5	0.31894 (14)	−0.11786 (14)	0.96113 (17)	0.0223 (3)	
C6	0.19710 (14)	−0.11148 (14)	1.03019 (16)	0.0223 (3)	
H6A	0.1526	−0.1831	1.0148	0.027*	
H6B	0.2136	−0.1068	1.1227	0.027*	
C7	0.11359 (13)	−0.01439 (13)	0.99450 (16)	0.0186 (3)	
H7	0.0555	0.0077	1.0636	0.022*	
C8	0.06109 (13)	−0.00524 (14)	0.86426 (16)	0.0204 (3)	
C9	0.10022 (14)	0.20144 (15)	0.93809 (17)	0.0230 (4)	
H9	0.0144	0.1915	0.9687	0.028*	
C10	0.17182 (18)	0.25947 (16)	1.04511 (19)	0.0333 (4)	
H10A	0.2567	0.2704	1.0183	0.050*	
H10B	0.1698	0.2120	1.1216	0.050*	
H10C	0.1348	0.3331	1.0639	0.050*	
C11	0.09512 (18)	0.27444 (16)	0.8189 (2)	0.0338 (4)	
H11A	0.0558	0.3467	0.8390	0.051*	
H11B	0.0481	0.2353	0.7529	0.051*	
H11C	0.1782	0.2883	0.7880	0.051*	
C12	0.35564 (17)	−0.23654 (15)	0.92405 (19)	0.0303 (4)	
H12A	0.4327	−0.2342	0.8766	0.045*	
H12B	0.2918	−0.2700	0.8706	0.045*	
H12C	0.3662	−0.2824	1.0010	0.045*	

### Atomic displacement parameters (Å^2^)

**Table:**

	*U*^11^	*U*^22^	*U*^33^	*U*^12^	*U*^13^	*U*^23^
Cl1	0.01859 (18)	0.0338 (2)	0.0541 (3)	0.00717 (17)	−0.0056 (2)	0.0017 (2)
Cl2	0.0291 (2)	0.0298 (2)	0.0332 (2)	0.00478 (17)	−0.00942 (19)	−0.00751 (19)
Cl3	0.01775 (18)	0.0357 (3)	0.0325 (2)	0.00250 (16)	−0.00432 (19)	0.00241 (19)
Cl4	0.0297 (2)	0.0359 (3)	0.0239 (2)	0.00577 (17)	−0.00508 (17)	−0.00837 (18)
C1	0.0177 (7)	0.0225 (8)	0.0161 (8)	0.0025 (6)	0.0012 (6)	0.0021 (6)
C2	0.0202 (8)	0.0252 (8)	0.0241 (9)	0.0021 (6)	0.0043 (7)	0.0065 (7)
C3	0.0183 (7)	0.0276 (9)	0.0227 (9)	0.0034 (6)	0.0030 (6)	0.0003 (7)
C4	0.0166 (7)	0.0250 (9)	0.0304 (10)	0.0056 (6)	−0.0019 (7)	−0.0004 (8)
C5	0.0203 (7)	0.0220 (8)	0.0247 (8)	0.0039 (6)	−0.0024 (6)	−0.0004 (7)
C6	0.0209 (7)	0.0241 (9)	0.0220 (8)	0.0006 (6)	−0.0001 (6)	0.0028 (7)
C7	0.0171 (7)	0.0214 (8)	0.0173 (8)	0.0004 (5)	0.0001 (6)	0.0008 (6)
C8	0.0166 (7)	0.0249 (8)	0.0198 (9)	0.0030 (6)	−0.0007 (6)	−0.0003 (7)
C9	0.0197 (7)	0.0236 (9)	0.0256 (9)	0.0049 (7)	0.0014 (6)	0.0022 (7)
C10	0.0332 (10)	0.0255 (9)	0.0411 (12)	0.0068 (8)	−0.0033 (8)	−0.0080 (8)
C11	0.0317 (10)	0.0299 (10)	0.0399 (12)	0.0072 (8)	0.0031 (7)	0.0124 (9)
C12	0.0295 (9)	0.0254 (10)	0.0359 (11)	0.0061 (7)	−0.0030 (8)	−0.0046 (8)

### Geometric parameters (Å, º)

**Table:**

Cl1—C4	1.7707 (15)	C6—C7	1.514 (2)
Cl2—C4	1.7547 (19)	C6—H6A	0.9900
Cl3—C8	1.7689 (14)	C6—H6B	0.9900
Cl4—C8	1.7648 (17)	C7—C8	1.488 (2)
C1—C8	1.507 (2)	C7—H7	1.0000
C1—C7	1.525 (2)	C9—C11	1.522 (2)
C1—C2	1.526 (2)	C9—C10	1.532 (3)
C1—C9	1.532 (2)	C9—H9	1.0000
C2—C3	1.515 (2)	C10—H10A	0.9800
C2—H2A	0.9900	C10—H10B	0.9800
C2—H2B	0.9900	C10—H10C	0.9800
C3—C4	1.493 (2)	C11—H11A	0.9800
C3—C5	1.529 (2)	C11—H11B	0.9800
C3—H3	1.0000	C11—H11C	0.9800
C4—C5	1.504 (2)	C12—H12A	0.9800
C5—C12	1.510 (2)	C12—H12B	0.9800
C5—C6	1.520 (2)	C12—H12C	0.9800
			
C8—C1—C7	58.76 (10)	C8—C7—C6	121.06 (14)
C8—C1—C2	116.86 (14)	C8—C7—C1	60.00 (11)
C7—C1—C2	118.66 (13)	C6—C7—C1	124.20 (13)
C8—C1—C9	117.56 (13)	C8—C7—H7	113.7
C7—C1—C9	118.21 (13)	C6—C7—H7	113.7
C2—C1—C9	115.19 (13)	C1—C7—H7	113.7
C3—C2—C1	117.11 (13)	C7—C8—C1	61.24 (11)
C3—C2—H2A	108.0	C7—C8—Cl4	119.49 (11)
C1—C2—H2A	108.0	C1—C8—Cl4	119.14 (11)
C3—C2—H2B	108.0	C7—C8—Cl3	118.56 (11)
C1—C2—H2B	108.0	C1—C8—Cl3	121.04 (11)
H2A—C2—H2B	107.3	Cl4—C8—Cl3	110.02 (9)
C4—C3—C2	121.99 (14)	C11—C9—C1	111.10 (14)
C4—C3—C5	59.69 (11)	C11—C9—C10	111.62 (16)
C2—C3—C5	123.80 (13)	C1—C9—C10	111.99 (13)
C4—C3—H3	113.7	C11—C9—H9	107.3
C2—C3—H3	113.7	C1—C9—H9	107.3
C5—C3—H3	113.7	C10—C9—H9	107.3
C3—C4—C5	61.33 (11)	C9—C10—H10A	109.5
C3—C4—Cl2	119.73 (11)	C9—C10—H10B	109.5
C5—C4—Cl2	119.31 (12)	H10A—C10—H10B	109.5
C3—C4—Cl1	118.71 (12)	C9—C10—H10C	109.5
C5—C4—Cl1	120.02 (12)	H10A—C10—H10C	109.5
Cl2—C4—Cl1	110.29 (9)	H10B—C10—H10C	109.5
C4—C5—C12	118.59 (14)	C9—C11—H11A	109.5
C4—C5—C6	117.36 (14)	C9—C11—H11B	109.5
C12—C5—C6	113.74 (14)	H11A—C11—H11B	109.5
C4—C5—C3	58.98 (11)	C9—C11—H11C	109.5
C12—C5—C3	118.63 (15)	H11A—C11—H11C	109.5
C6—C5—C3	119.01 (13)	H11B—C11—H11C	109.5
C7—C6—C5	116.70 (14)	C5—C12—H12A	109.5
C7—C6—H6A	108.1	C5—C12—H12B	109.5
C5—C6—H6A	108.1	H12A—C12—H12B	109.5
C7—C6—H6B	108.1	C5—C12—H12C	109.5
C5—C6—H6B	108.1	H12A—C12—H12C	109.5
H6A—C6—H6B	107.3	H12B—C12—H12C	109.5
			
C8—C1—C2—C3	−66.2 (2)	C5—C6—C7—C8	64.6 (2)
C7—C1—C2—C3	1.2 (2)	C5—C6—C7—C1	−8.1 (2)
C9—C1—C2—C3	149.62 (15)	C2—C1—C7—C8	−105.70 (16)
C1—C2—C3—C4	−73.4 (2)	C9—C1—C7—C8	106.78 (15)
C1—C2—C3—C5	−0.8 (2)	C8—C1—C7—C6	109.15 (17)
C2—C3—C4—C5	113.23 (16)	C2—C1—C7—C6	3.4 (2)
C2—C3—C4—Cl2	3.9 (2)	C9—C1—C7—C6	−144.07 (16)
C5—C3—C4—Cl2	−109.28 (14)	C6—C7—C8—C1	−114.21 (16)
C2—C3—C4—Cl1	−136.23 (13)	C6—C7—C8—Cl4	−5.1 (2)
C5—C3—C4—Cl1	110.53 (15)	C1—C7—C8—Cl4	109.13 (13)
C3—C4—C5—C12	108.00 (18)	C6—C7—C8—Cl3	134.01 (13)
Cl2—C4—C5—C12	−142.04 (15)	C1—C7—C8—Cl3	−111.78 (14)
Cl1—C4—C5—C12	−0.4 (2)	C2—C1—C8—C7	108.76 (15)
C3—C4—C5—C6	−109.03 (16)	C9—C1—C8—C7	−107.88 (16)
Cl2—C4—C5—C6	0.9 (2)	C7—C1—C8—Cl4	−109.69 (14)
Cl1—C4—C5—C6	142.53 (14)	C2—C1—C8—Cl4	−0.9 (2)
Cl2—C4—C5—C3	109.95 (14)	C9—C1—C8—Cl4	142.43 (13)
Cl1—C4—C5—C3	−108.44 (15)	C7—C1—C8—Cl3	107.83 (14)
C2—C3—C5—C4	−110.31 (18)	C2—C1—C8—Cl3	−143.41 (13)
C4—C3—C5—C12	−107.94 (17)	C9—C1—C8—Cl3	0.0 (2)
C2—C3—C5—C12	141.75 (17)	C8—C1—C9—C11	−86.75 (17)
C4—C3—C5—C6	106.25 (17)	C7—C1—C9—C11	−154.17 (14)
C2—C3—C5—C6	−4.1 (2)	C2—C1—C9—C11	57.21 (19)
C4—C5—C6—C7	76.0 (2)	C8—C1—C9—C10	147.68 (16)
C12—C5—C6—C7	−139.28 (15)	C7—C1—C9—C10	80.26 (18)
C3—C5—C6—C7	8.1 (2)	C2—C1—C9—C10	−68.36 (19)
